# Diagnosing melanoma patients entering American Joint Committee on Cancer stage IV, C-reactive protein in serum is superior to lactate dehydrogenase

**DOI:** 10.1038/sj.bjc.6602043

**Published:** 2004-07-27

**Authors:** M Deichmann, B Kahle, K Moser, J Wacker, K Wüst

**Affiliations:** 1 Department of Dermatology, Heidelberg University Clinics, Voßstraße 2, 69115 Heidelberg, Germany; 2 Institute for Medical Biometry, University of Heidelberg, Im Neuenheimer Feld 305, 69120 Heidelberg, Germany

**Keywords:** melanoma, American Joint Committee on Cancer stage IV, serum marker, tumour marker, lactate dehydrogenase (LDH), C-reactive protein (CRP)

## Abstract

Lactate dehydrogenase (LDH) in serum has recently been introduced into the American Joint Committee on Cancer (AJCC) staging system for cutaneous melanoma because of its prognostic value. We hypothesised LDH to be of value in discriminating melanoma patients entering AJCC stage IV from patients staying in AJCC stages I, II or III. Lactate dehydrogenase was compared to the acute phase protein C-reactive protein (CRP), which we observed to reflect the course of melanoma metastasis in a previous report. In this prospective study, we measured LDH and CRP in the serum of 91 consecutive melanoma patients progressing into AJCC stage IV in comparison to 125 patients staying in AJCC stages I, II or III. Comparing distributions of the parameters by median values and quartiles by Mann–Whitney test, LDH was not significantly elevated in patients entering AJCC stage IV melanoma (*P*=0.785), whereas CRP was (*P*<0.001). Analysing the sensitivity and the specificity jointly by the areas under the receiver operating characteristics curves (ROC-AUC), LDH did not discriminate between the defined groups of patients (AUC=0.491; 95% confidence interval, 0.410, 0.581), whereas CRP did (AUC=0.933; 95% confidence interval, 0.900, 0.966; *P*<0.001). Upon logistic regression analysis to calculate the ROC-AUC values upon the predictive probabilities, LDH provided no additional information to CRP. Choosing a cutoff point of 3.0 mg l^−1^, CRP yielded a sensitivity of 0.769 together with a specificity of 0.904 in diagnosing AJCC stage IV entry. Altogether, for first diagnosing AJCC stage IV melanoma, CRP is the superior serum marker when compared to the conventional LDH.

Melanomas are one of the most aggressive of the skin cancers and have shown a dramatic increase in incidence over the past decades (Rigel *et al*, 1996). Following excision of the primary tumour, patients are followed up in regular intervals in order to detect distant metastases early and thus to, if possible, get patients free of disease by surgery or to monitor the course of metastasis during chemotherapy. In these follow-up examinations, serum parameters, such as S100*β* or melanoma-inhibiting activity (MIA), have been evaluated for discriminating progressive from nonprogressive American Joint Committee on Cancer (AJCC) stage IV disease; unfortunately, these markers did not provide additional significant information to the conventional lactate dehydrogenase (LDH; Deichmann *et al*, 1999), which is the most predictive independent factor of diminished survival upon multivariate analysis, even after accounting for site and number of metastases (Sirott *et al*, 1993; Eton *et al*, 1998; Franzke *et al*, 1998; Keilholz *et al*, 1998). Lactate dehydrogenase has not only prognostic value in melanoma but also in further tumours, for example, small-cell and non-small-cell lung cancer, Hodgkin's and Non-Hodgkin's lymphoma or prostate cancer (Sirott *et al*, 1993). In view of the favourite data in melanoma, LDH has even been introduced into the new classification system of the AJCC (Balch *et al*, 2001).

Unexpectedly, prospective studies with statistically assessable cohorts of patients have not yet been conducted to evaluate the value of LDH in discriminating melanoma patients entering AJCC stage IV from patients staying in AJCC stages I, II or III during follow up. We therefore initiated the present study and measured LDH in a consecutive series of 91 patients with first diagnosis of AJCC stage IV melanoma in comparison to 125 patients staying in AJCC stages I, II or III. In parallel, we also measured the acute phase protein C-reactive protein (CRP), which we previously found to reflect the course of distant metastasis (Deichmann *et al*, 2000; Deichmann *et al*, 2001). Distributions of the parameters by median values and quartiles were compared by Fisher's exact test and the Mann–Whitney test. The sensitivity and the specificity were jointly analysed by the areas under the receiver operating characteristics curves (ROC-AUC).

## PATIENTS AND LABORATORY ANALYSES

Between April 1999 and April 2004, 91 consecutive patients with metastatic melanoma were entered onto the study. All patients had histologically confirmed melanoma (20 superficial spreading, 12 nodular, five acral lentiginous, 24 cutaneous melanomas not further classified, nine uveal, nine mucosal, 12 no primary tumour). The time point of entry into the study was the first diagnosis of clinical stage IV according to the AJCC criteria (Balch *et al*, 2001). Metastases were diagnosed by clinical examination, chest X-rays, ultrasound, computed axial tomography scans and nuclear magnetic resonance imaging. A total of 11 patients suffered from distant skin, subcutaneous or lymph node metastases, 19 patients had lung metastases, and other visceral metastases were found in 61 patients.

Blood samples were taken at the time of the first diagnosis of AJCC stage IV melanoma. Lactate dehydrogenase was tested on a Beckman LX20 instrument (Beckman Instruments, Gagny, France), and CRP was assayed by a rate immunonephelometric technique on a protein system analyser (BN2 Array; Dode-Behring, Munich, Germany).

In total, 125 consecutive melanoma patients, not being diagnosed as AJCC stage IV in follow-up examinations, entered onto the study as cohort for comparison. Again, these patients had histologically confirmed melanoma (53 superficial spreading, 26 nodular, 10 acral lentiginous, four lentiginous, 31 cutaneous melanomas not further classified, one no primary tumour). At the time point when blood was drawn, these patients were diagnosed as melanoma stage I (21 patients), stage II (49 patients) and stage III (55 patients) according the AJCC criteria (Balch *et al*, 2001).

### Statistical analyses

All statistics and figures were computed with the statistical software SPSS (SPSS Inc., Release 10.0.7, Chicago, IL, USA). We report two-tailed statistics throughout, a *P*-value smaller than 0.05 (*P*<0.05) was considered significant. Two-sample comparisons were performed by Fisher's exact test (sex) and the Mann–Whitney test (age, LDH, CRP). The receiver operating characteristics (ROC) analysis was used to evaluate the ability of the conventional serum marker LDH and the CRP serum values to discriminate between patients entering AJCC stage IV (IV+) from those staying in AJCC stages I, II or III (IV−). The primary result of the ROC analysis was the area under the curve (AUC), which is a measure for the effect size. The AUC corresponds to the probability that a randomly chosen pair from (IV+, IV−) is diagnosed correctly. An AUC of 0.5 or below indicates that a diagnostic tool does not discriminate better than chance between IV+ and IV− patients, an AUC of 1 indicates a perfect discriminability. The ROC analysis can be conducted choosing the values of the markers themselves as cutoff points or, alternatively, the predictive probabilities that result from a logistic regression with the markers as independent variables. To compare the discriminability of the marker CRP alone to the discriminability of the marker CRP in combination with the conventionally used marker LDH, we conducted two logistic regressions: one on CRP alone and one on CRP in combination with LDH. The null hypothesis *H*_0_ : *AUC*_CRP_ − *AUC*_CRP + LDH_ = 0 against *H*_A_ : *AUC*_CRP_ − *AUC*_CRP + LDH_ ≠ 0 was then tested using the test statistic derived for correlated AUCs by Hanley and McNeil (1983). For the diagnostic procedure proven to be superior, sensitivities and cutoff points are given for the specificities of 0.8, 0.85 and 0.9.

## RESULTS

Comparing the melanoma patients entering AJCC stage IV with those staying in AJCC stages I, II and III, both groups did not differ significantly in sex and age (Table 1

**Table 1:** Median, lower and upper quartiles, and *P-*values according to two-sample comparisons

	**Melanoma AJCC stage IV**		**Melanoma not AJCC stage IV**		
	**Median**	**Lower and upper quartiles**	**Median**	**Lower and upper quartiles**	***P*^a^**
No. of patients		91		125	
Male (%)		59.3		55.2	0.580
Age (years)	59	51–69	58	46–69	0.397
LDH (U l^−1^)	197	150–328	208	185.5–235.5	0.785
CRP (mg l^−1^)	14.8	3.6–44.2	<2	<2–<2^b^	<0.001

a Fisher's exact test (sex) and Mann–Whitney test.

b Maximum: 7.7, 95% quantile: 5.47.

LDH=lactate dehydrogenase; CRP=C-reactive protein.

). Describing their distributions by median values and quartiles using the Mann–Whitney test, serum CRP was markedly elevated in patients entering AJCC stage IV melanoma (*P*<0.001), whereas LDH was not (*P*=0.785; Table 1).

**Figure 1:**
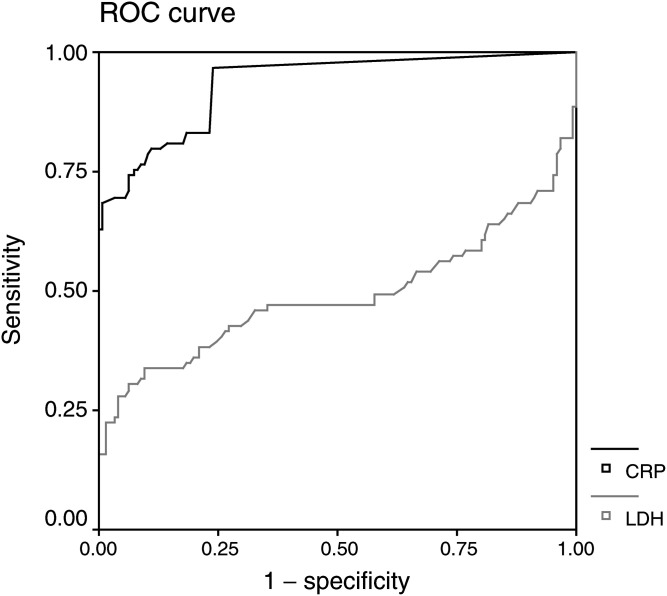
Receiver operating characteristic (ROC) curves for the serum parameters LDH (grey line) and CRP (black line). As the area under the curve (AUC) is larger for CRP, this marker is better in discriminating melanoma patients entering AJCC stage IV from patients staying in stages I, II or III in follow-up examinations.

Estimates for both sensitivity and specificity in separating patients entering AJCC stage IV melanoma from those staying in AJCC stages I, II or III were obtained using ROC analysis. The figure already indicates that the ability of discrimination of the marker CRP is clearly better than one of the marker LDH (Figure 1

). The AUC analysis confirms that LDH (AUC=0.491; 95% confidence interval (0.410, 0.581)) did not discriminate significantly (*P*=0.823), whereas CRP did for the considered population (AUC=0.933; 95% confidence interval (0.900, 0.966); *P*<0.001).

**Figure 2:**
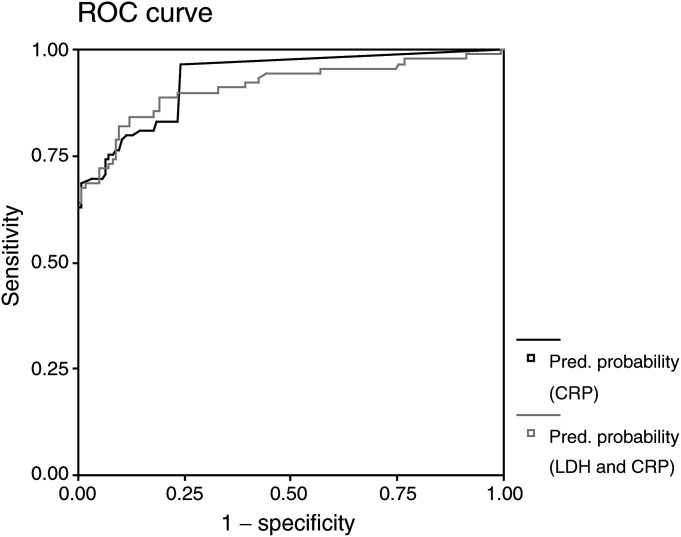
Receiver operating characteristic (ROC) curves for the parameter CRP (black line) in comparison to the combination of CRP and LDH (grey line) in discriminating melanoma patients entering AJCC stage IV from patients staying in stages I, II or III in follow-up examinations. The area under the curve (AUC) of CRP alone (0.933) is larger than the AUC of CRP in combination with LDH (0.913); still this difference was not statistically significant.

Next, we compared CRP to the combination of CRP and LDH in the diagnosis of AJCC stage IV melanoma entry. We conducted logistic regressions on CRP alone and on CRP and LDH together. Denoting by *π* the probability that a patient has entered AJCC stage IV, the logistic regression on CRP yielded *π*=1/(1+exp(−(−2.906+0.575 X_crp_)), where *X*_crp_ denoted the amount of CRP measured. The logistic regression on CRP and LDH yielded *π*=1/(1+exp(−(−1.7147+0.626*X*_crp_−0.006*X*_ldh_)), where *X*_crp_ and *X*_ldh_ denoted the amount of CRP and LDH measured, respectively. Using the predicted probabilities for a patient being diagnosed to enter AJCC stage IV as cutoff points, the ROC curve of CRP alone diagnosing entry AJCC stage IV was then compared with the ROC curve of CRP and LDH together (Figure 2

). The figure already indicates that CRP alone is slightly better than the combination of CRP and LDH. We compared the corresponding AUCs of CRP alone which resulted to be 0.933 (standard deviation (s.d.), 0.017; 95% confidence interval, 0.900, 0.966) and CRP combined with LDH which was 0.913 (0.022; 95% confidence interval, 0.869, 0.957).

To test whether the AUC of CRP in combination with LDH is significantly larger than the AUC of CRP alone, we applied the Hanley–McNeil test statistic, calculating the correlation using Kendall's *τ* of the predictive probabilities of CRP and CRP in combination with LDH for patients with and without AJCC stage IV diagnosis, respectively. This lead to correlations of 0.475 for patients with distant metastases and 0.917 for patients without distant metastases. Pooling these values gave an average correlation of 0.696. Together with the mean AUCs of 0.923, we derived the correlation coefficient *r* between the two AUC from the tables given by Hanley and McNeil (1983), which was 0.60. The Hanley–McNeil test statistic 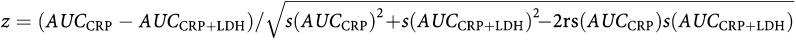 with *AUC*_CRP_ and *AUC*_CRP+LDH_ being the areas under the curve for CRP and the combination of CRP and LDH and *s*(*AUC*_CRP_) and *s*(*AUC*_CRP+LDH_) their s.d.'s yielded a value of 1.11, which can be compared to the critical values of the normal distribution. Hence, the null hypothesis could not be rejected: the AUC of CRP in combination with LDH could not be proven to be significantly larger than the AUC of CRP alone.

The marker CRP thus was proven to discriminate better than LDH between patients entering AJCC stage IV from patients staying in AJCC stages I, II or III. To reach a specificity of about 0.8, one could either choose a cutoff point of 2.1 leading to a specificity of 0.792 or a cutoff point of 2.2 leading to a specificity of 0.816. Both cutoff points corresponded to sensitivities of 0.835. For a specificity of about 0.85, the cutoff points of 2.4 or 2.5 yielded specificities of 0.832 and 0.856, respectively. Both cutoff points corresponded to sensitivities of 0.813. With a cutoff point of 2.9, a specificity of 0.896 was reached together with a sensitivity of 0.791, while a cutoff point of 3.0 yielded a specificity of 0.904 together with a sensitivity of 0.769.

## DISCUSSION

For discriminating melanoma patients entering AJCC stage IV from patients staying in AJCC stages I, II or III during follow up, LDH is not suitable, regardless of its proven prognostic value in AJCC stage IV (Sirott *et al*, 1993; Eton *et al*, 1998; Franzke *et al*, 1998; Keilholz *et al*, 1998; Balch *et al*, 2001). Unexpectedly, CRP resulted as the more favourite serum marker: This acute phase protein was superior to LDH and contributed significantly in discriminating melanoma patients entering AJCC stage IV from patients staying in AJCC stages I, II or III.

C-reactive protein was introduced in this study as elevated levels in serum have been associated with shortened survival in metastatic melanoma patients and resistance to interleukin-2 therapy (Tartour *et al*, 1994; Mouawad *et al*, 1996; Tartour *et al*, 1996). In line with this report, we previously observed high CRP levels to indicate progression of distant metastases during therapy (Deichmann *et al*, 2000). As a nonspecific acute phase response to almost all forms of inflammation (Pepys, 1995), CRP is synthesised in hepatocytes in response to cytokines such as mainly interleukin-6 (Il-6; Moshage *et al*, 1988; Castell *et al*, 1989; Kishimoto, 1989; Klein *et al*, 1991; Hirano, 1992). As melanoma cells are known to produce Il-6 (Colombo *et al*, 1992; Lee *et al*, 1992; Lu and Kerbel 1993; Mattei *et al*, 1994; Francis *et al*, 1996), serum CRP might reflect Il-6 production from the tumour. However, tumour-specific immune interaction with T cells, macrophages or monocytes might also be responsible for elevated Il-6 levels, as these cell are known to produce this cytokine, too. Further major sources of Il-6 may be surrounding fibroblasts or endothelial cells (Kishimoto, 1989). *In vivo*, CRP reflects Il-6 secretion (Klein *et al*, 1991) supported by high correlation between serum CRP and Il-6 concentrations in melanoma patients (Moshage *et al*, 1988; Castell *et al*, 1989; Kishimoto, 1989; Klein *et al*, 1991; Hirano, 1992; Tartour *et al*, 1996; Deichmann *et al*, 2000).

As Il-6 is known to inhibit the activity of lipoprotein lipase, a key regulatory enzyme for triglyceride clearance from plasma, increased levels of its surrogate CRP were positively correlated with the paraneoplastic inflammatory syndrome, leading to weight loss cachexia (Mahmoud and Rivera, 2002). Besides melanoma (Tartour *et al*, 1994; Tartour *et al*, 1996), high CRP serum levels are known to indicate shorter median survival in patients suffering from other tumours such as renal cell carcinoma (Blay *et al*, 1992) or colorectal carcinoma (Gough *et al*, 1996; Nozoe *et al*, 1998; Mahmoud and Rivera, 2002). Thus, CRP is not exclusively increased in melanoma. This protein is produced as a nonspecific acute phase response to almost all forms of inflammation (Pepys, 1995).

Altogether, LDH did not discriminate melanoma patients entering AJCC stage IV from patients staying in AJCC stages I, II or III in follow-up examinations, whereas CRP did upon ROC-AUC analysis. Choosing a cutoff point of 3.0 mg l^−1^, CRP yielded a sensitivity of 77% together with a specificity of 90% in diagnosing AJCC stage IV entry. Following excision of the primary melanoma, CRP measurement may contribute to earlier detection of distant metastasis, possibly getting some patients free of disease by surgery or radiation. We therefore recommend the routine measurement of CRP in follow-up examinations of melanoma patients, which is a simple and cheap test and provides valuable information.
